# Single-Cell Transcriptome Reveals the Metabolic and Clinical Features of a Highly Malignant Cell Subpopulation in Pancreatic Ductal Adenocarcinoma

**DOI:** 10.3389/fcell.2022.798165

**Published:** 2022-02-18

**Authors:** Yangyang Fang, Shunjie Pei, Kaizhao Huang, Feng Xu, Guangxin Xiang, Linhua Lan, Xiaoqun Zheng

**Affiliations:** ^1^ Department of Laboratory Medicine, The Second Affiliated Hospital and Yuying Children's Hospital of Wenzhou Medical University, Wenzhou, China; ^2^ School of Laboratory Medical and Life Science, Wenzhou Medical University, Wenzhou, China; ^3^ Key Laboratory of Laboratory Medicine, School of Laboratory Medicine and Life Science, Wenzhou Medical University, Wenzhou, China; ^4^ Key Laboratory of Diagnosis and Treatment of Severe Hepato-Pancreatic Diseases of Zhejiang Province, The First Affiliated Hospital of Wenzhou Medical University, Wenzhou, China

**Keywords:** single-cell transcriptome, pancreatic ductal adenocarcinoma, malignant, tumor metabolic, glycolysis

## Abstract

**Background:** Pancreatic ductal adenocarcinoma (PDAC) is a malignant tumor with a high mortality rate. PDAC exhibits significant heterogeneity as well as alterations in metabolic pathways that are associated with its malignant progression. In this study, we explored the metabolic and clinical features of a highly malignant subgroup of PDAC based on single-cell transcriptome technology.

**Methods:** A highly malignant cell subpopulation was identified at single-cell resolution based on the expression of malignant genes. The metabolic landscape of different cell types was analyzed based on metabolic pathway gene sets. *In vitro* experiments to verify the biological functions of the marker genes were performed. PDAC patient subgroups with highly malignant cell subpopulations were distinguished according to five glycolytic marker genes. Five glycolytic highly malignant-related gene signatures were used to construct the glycolytic highly malignant-related genes signature (GHS) scores.

**Results:** This study identified a highly malignant tumor cell subpopulation from the single-cell RNA sequencing (scRNA-seq) data. The analysis of the metabolic pathway revealed that highly malignant cells had an abnormally active metabolism, and enhanced glycolysis was a major metabolic feature. Five glycolytic marker genes that accounted for the highly malignant cell subpopulations were identified, namely, *EN O 1*, *LDHA*, *PKM*, *PGK1*, and *PGM1*. An *in vitro* cell experiment showed that proliferation rates of PANC-1 and CFPAC-1 cell lines decreased after knockdown of these five genes. Patients with metabolic profiles of highly malignant cell subpopulations exhibit clinical features of higher mortality, higher mutational burden, and immune deserts. The GHS score evaluated using the five marker genes was an independent prognostic factor for patients with PDAC.

**Conclusion:** We revealed a subpopulation of highly malignant cells in PDAC with enhanced glycolysis as the main metabolic feature. We obtained five glycolytic marker gene signatures, which could be used to identify PDAC patient subgroups with highly malignant cell subpopulations, and proposed a GHS prognostic score.

## Introduction

Pancreatic cancer has a high mortality rate, with a 5-year survival rate of only 10%. Pancreatic ductal adenocarcinoma (PDAC) contributes to approximately 90% of all pancreatic malignancies (Siegel et al., 2021). Its high mortality rate is mainly owing to its highly aggressive nature that exhibits significant heterogeneity (Rodriguez-Aznar et al., 2019). Therefore, identifying highly malignant subtypes will help to select patients who will benefit most from neoadjuvant therapy before surgery.

Metabolic reprogramming has been recognized as a common feature of cancer. Tumor cells adopt several metabolic pathways using reprogramming to meet the large energy requirements of cell growth (Vander Heiden and DeBerardinis, 2017). In PDAC, the tumor develops in a highly fibrotic and connective tissue-proliferating microenvironment that causes compression of blood vessels and insufficient blood perfusion (Stylianopoulos et al., 2012). Therefore, the glucose uptake rate of PDAC cells is mostly moderate. However, it demonstrates a high growth rate in a nutrient-deficit environment (Halbrook and Lyssiotis, 2017). These features reflect the critical role of metabolism in PDAC progression. Therefore, more therapeutic modalities focusing on metabolic targets of PDAC have been developed in recent years (Boudreau et al., 2016; Protopopova et al., 2016); however, there is a lack of suitable biomarkers to differentiate metabolic subtypes.

Single-cell RNA sequencing (scRNA-seq), with its characteristics of analyzing transcriptomic information at the individual cell level, is often used to discover new cell subtypes, reveal cell heterogeneity, monitor the dynamic process of disease development, etc. (González-Silva et al., 2020). Metabolic gene expression can help us to improve our understanding of metabolic pathway activity (Lee et al., 2012). In contrast to traditional bulk RNA-seq, the characteristics of scRNA-seq will gain an insight into the metabolic heterogeneity in malignant cells.

In this study, the metabolic and clinical features of a highly malignant cell subpopulation were investigated in PDAC using scRNA-seq data, bulk RNA sequencing (RNA-seq) data, and cell function experiments. The highly malignant cell subpopulation was identified to have a high degree of glycolysis. In addition, five glycolytic marker genes were used to differentiate patient metabolic subtypes and predict tumor progression. Therefore, we provided an insight into metabolic heterogeneity in PDAC.

## Materials and Methods

### Data Source

PDAC scRNA-seq datasets were downloaded from the Genome Sequence Archive (GSA) database (https://bigd.big.ac.cn/gsa), under accession number CRA001160 (Zhang et al., 2018). A total of 41,986 cells from 24 human pancreatic cancer tissues without preoperative radiotherapy were included in the analysis (Peng et al., 2019). The RNA-seq data and corresponding clinical records of PDAC were downloaded from the Cancer Genome Atlas (TCGA)-PAAD (n = 146) and GSE62452 (n = 130). Image data in the study were obtained from the Human Protein Atlas (HPA).

### scRNA-Seq Data Quality Control and Analysis

Single-cell RNA-seq matrices were filtered out for cells (<200 transcripts/cell, >10% mitochondrial genes) and genes (<10 cells/gene). We performed a subsequent analysis of the data using the Seurat R package (Hao et al., 2021). The data are pre-processed using standard steps (https://satijalab.org/seurat). Finally, single-cell clustering was visualized using t-distributed stochastic neighbor embedding (t-SNE) and Uniform Manifold Approximation and Projection (UMAP) (Wu et al., 2019). The transcriptome of quality filtered cells was further normalized using the scran package (Lun et al., 2016).

Copy number variation (CNV) scores were inferred from scRNA-seq data using the inferCNV R package (Version1.6.0, https://gitmarker.com/broadinstitute/inferCNV). Quantification of CNV scores of different cells was conducted using the hidden Markov model (HMM) (Tirosh et al., 2016). Normalized gene–cell matrices were used to calculate the metabolic pathway scores and malignancy scores for each cell. We referred to public methods for quantifying metabolic pathways in scRNA-seq data (Xiao et al., 2019). All related code was available on GitHub (GitHub, Inc., San Francisco, California) at https://gitmarker.com/LocasaleLab/Single-Cell-Metabolic-Landscape. Malignant genes were obtained from previous studies, while metabolic-related genes were excluded (Peng et al., 2019). The malignant score is the average expression level of malignant genes. Gaussian mixture models were estimated using the “mixtools” package (Version1.2.0, https://cran.r-project.org/web/packages/mixtools/index.html) using posterior probability to have soft assignment for each cell. Correlation matrices of significant correlations were plotted and visualized using the Corrplot function of the corrplot package. Enrichment analysis of marker genes for highly and lowly malignant cell subpopulations was done using compareCluster function in clusterProfiler (Wu et al., 2021). Enrichment pathways were ranked using false discovery rate (FDR) from lowest to highest. To distinguish differentially expressed genes in single-cell datasets, calculations were performed using the edgeR package of R software (Robinson et al., 2010). The relevant code to build the pseudo-bulk data was obtained at https://hbctraining.github.io/scRNA-seq/lessons/pseudobulk_DESeq2_scrnaseq.

### Bulk RNA-Seq Data Quality Control and Analysis

The count data of the bulk RNA-seq datasets were removed from the batch effect using the ComBat-seq function of the sva package (Zhang et al., 2020). Gene expression profiling interaction analysis (GEPIA, http://gepia.cancer-pku.cn) was used to analyze the mRNA expression of TCGA program and the Genotype-Tissue Expression (GTEx) data (Tang et al., 2019). Survival analysis was performed according to gene expression levels. The optimal cut-off point was determined using the R package survminer. Patients were reclassified using the umap package (Mcinnes and Healy, 2018). Immune cell infiltration scores were computed through the GSVA R package with method specification as single-sample Gene Set Enrichment Analysis (ssGSEA) (Hnzelmann et al., 2013). Gene sets signatures of anti-tumor and pro-tumor immune cells were obtained from a study mentioned in another article (Jia et al., 2018).

### Cell Culture

HEK293 cells, HPDE cells, and pancreatic cell lines, including PANC-1 and CFPAC-1 cells, were purchased from the Cell Bank of the Chinese Academy of Sciences (Shanghai, China). HEK293, HPDE, and PANC-1 cells were cultured in Dulbecco's Modified Eagle Medium, and CFPAC-1 cell line was cultured in Roswell Park Memorial Institute-1640 medium, both of which were supplemented with 10% fetal bovine serum (10099141, Gibco™) and antibiotics (100 U/ml penicillin and 100 μg/ml streptomycin, C0222, Beyotime) at 37°C, 5% CO_2_, respectively.

### Lentivirus Packaging for RNA Interfere

HEK293 cells were cultured for lentivirus packaging. Briefly, HEK293 cells were seeded into 6-cm dishes at a density of 2.5 × 10^6^ cells/dish and cultured in an incubator at 37°C with 5% CO_2_ overnight. Furthermore, cells were transfected with the following plasmids: Lipofectamine 2000 reagent (11668019, Invitrogen™): PXPAX2: PMD2G: target plasmid at a ratio of 10 μl:2 μg:1 μg:2 μg. Cell cultured medium was collected at 24, 48, and 72 h, and subsequently filtered using a 0.45-μm filter. Furthermore, PANC-1, CFPAC-1, and HPDE cells were seeded into 6-cm dishes, and prepared lentivirus solutions were added into corresponding dishes. After 48 h, the cell culture medium was replaced with a fresh medium supplemented with 2 μg/ml of puromycin (P8230, Solarbio). Successfully transfected cells were selected and confirmed using Western blot analysis with indicated antibodies. The lentiviral-based short hairpin RNA (shRNA) vector was collected from the Public Protein/Plasmid Library. The detailed information was as follows: pPLK/GFP + Puro-ENO1 shRNA (Catalogue number: 2023), pPLK/GFP + Puro-LDHA shRNA (Catalogue number: 3939), pPLK/GFP + Puro-PKM2 shRNA (Catalogue number: 5315), pPLK/GFP + Puro-PGK1 shRNA (Catalogue number: 5230) and pPLK/GFP + Puro-PGM1 shRNA (Catalogue number: 5236).

### Measurement of Cell Proliferation

To evaluate the effects of knockdown of glycolytic enzymes on PDAC cell proliferation, cell viability was measured using a CCK-8 cell proliferation kit. For example, shCont, shENO1#1, and shENO1#1 PDAC cells were seeded into 96-well cell culture plates at a density of 5 × 10^3^ cells/well and incubated with CCK-8 working solution at 37°C and 5% CO_2_ for 2 h. The optical density (OD) value at 450 nm was measured using a micro-plate reader. The relative cell proliferation rate was represented as mean ± standard deviation (SD). Each sample included six replicates. The protocol was also performed to evaluate the effects of *LDHA*, *PKM2*, *PGK1*, and *PGM1* downregulation on PDAC cell as well as pancreatic normal ductal cell proliferation.

### Development of the Prognostic Glycolysis Highly Malignant Related Genes Signature

Referring to the public method (Hao et al., 2018), we combined the hazard ratio (HR) of five key glycolytic genes with the standard estimations (SE) as the prognostic glycolytic gene weights to generate the glycolytic highly malignant-related genes signature (GHS) prognostic score, which has the advantage of reducing the effect of sample size on the weight of each gene. The GHS score of the sample is given by the following equation:

$$\mathrm{GHS}=\sum_{i\mathrm{=1}}^{5}\frac{HR_{i}\mathrm{-1}}{\mathrm{SE}\left(HR_{i}\right)}\mathrm{*gene}\left(i\right)$$

Lastly, the prognostic value of the GHS score was assessed using single-factor and multi-factor Cox proportional hazard analysis. Predictive performance was assessed using receiver operating characteristic (ROC) curves.

### Statistical Analysis

The chi-square test was used to investigate the differences in clinicopathological features among the three subtypes. ANOVA was used to identify the expression levels of the three subtypes. The *t*-test was used to investigate the differences between the two groups. Correlation analysis was performed using Pearson's correlation coefficient. Statistical analysis was performed in R (version 4.0.4) (Rdct, 2005). The experimental data in the article were analyzed using GraphPad Prism 9.0 software. A *p*-value < 0.05 was considered statistically significant.

## Results

### Single-Cell RNA Sequencing Identifies a Highly Malignant Subpopulation of Ductal Cells

**FIGURE 1:**
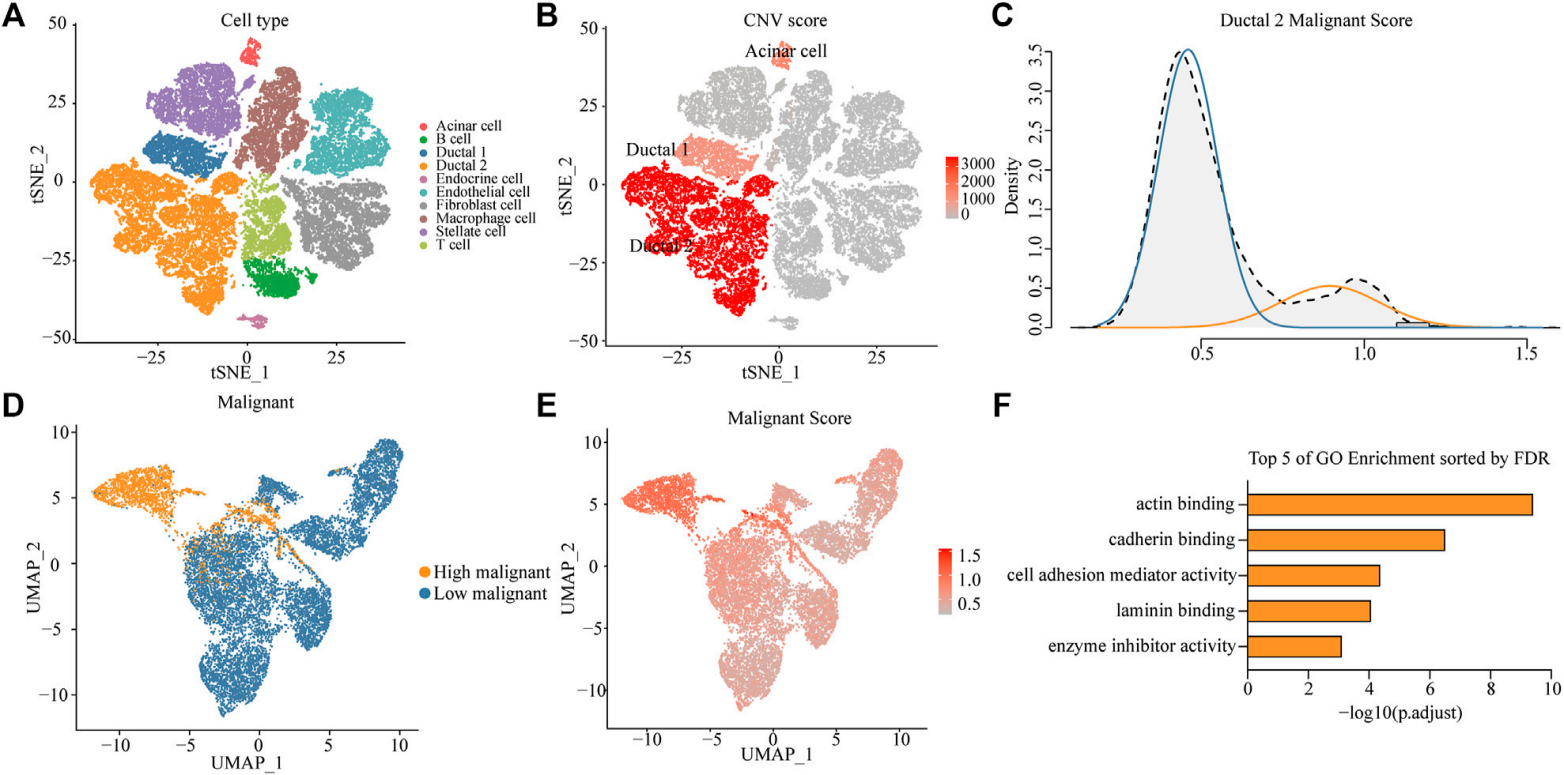
Identifying a highly malignant cell subpopulation in PDAC. **(A)** t-SNE dimensional reduction of gene expression in 40,986 cells showing major cell types in 24 patients with PDAC. **(B)** Copy number variation (CNV) scores of ductal 1, ductal 2, and acinar cells via t-SNE analysis. **(C)** A density graph of malignant scores in ductal 2 cells. **(D)** UMAP plots comparing highly malignant and lowly malignant cells in ductal 2 cells. **(E)** UMAP plots of corresponding malignant scores levels [scaled from low (gray) to high (red)]. **(F)** Bar chart of highly malignant cell biological process (BP) GO terms (at most five) ranked by the FDR (*p* < 0.05).

High aggressiveness and heterogeneity of PDAC lead to extremely low survival rates of patients. scRNA-seq data were obtained from 24 patients with PDAC, and 10 different clusters including acinar cells, B cells, ductal 1 cells, ductal 2 cells, endothelial cells, endocrine cells, fibroblasts, macrophages, stellate cells, and T cells were finally identified by standardizing the single-cell processing steps. The marker genes for each cell cluster were consistent with known cellular markers (Figure 1A and Supplementary Figure S1A). To further validate the assignment of cell subpopulations, we performed an inferred gene copy number analysis using the InferCNV R package. The CNV scores of ductal and acinar cells were predicted using the HMM method. Ductal 2 cells had significantly higher CNV scores than those of ductal 1 cells (Figure 1B and Supplementary Figure S1B), which suggested that ductal 2 cells were a malignant cell subpopulation.

To investigate the possibility of distinctions among malignant cells in terms of the degree of malignancy, we collated 136 genes involved in the malignant progression of PDAC (Supplementary Table S1). After normalization of scRNA-seq data (Supplementary Figure S1C), we scored malignant ductal 2 cells based on the collected PDAC malignant genes and found a bimodal distribution of malignancy scores for ductal 2 cells. This finding implied that some cells in PDAC exhibited features of highly malignant tendency. After a Gaussian fit of the data, we classified the malignant ductal cells into a high- and low-malignancy group according to the malignant score (Figure 1C).

### Unique Gene Expression Profiles of Highly Malignant Cell Subpopulations

To investigate the existence of a unique expression profile for the subpopulation of highly malignant cells, we projected ductal 2 cells to a two-dimensional plane using the UMAP dimensionality reduction method. Moreover, highly malignant cells were distinguished (Figure 1D), suggesting that highly malignant tumor cells had a unique expression profile (Supplementary Figure S2A). Gene Ontology (GO) terms of the highly malignant cell subpopulation were identified using the compareCluster function in the R package clusterProfiler. GO enrichment analysis revealed that the top five pathways associated with highly malignant cells were mainly associated with actin-binding, cadherin-binding, cell adhesion mediator activity, laminin-binding, and enzyme-inhibitor activity (Figure 1F and Supplementary Table S2). All terms were recognized to be strongly associated with cancer progression and metastasis.

### Metabolic Reprogramming of Highly Malignant Cell Subpopulations

**FIGURE 2:**
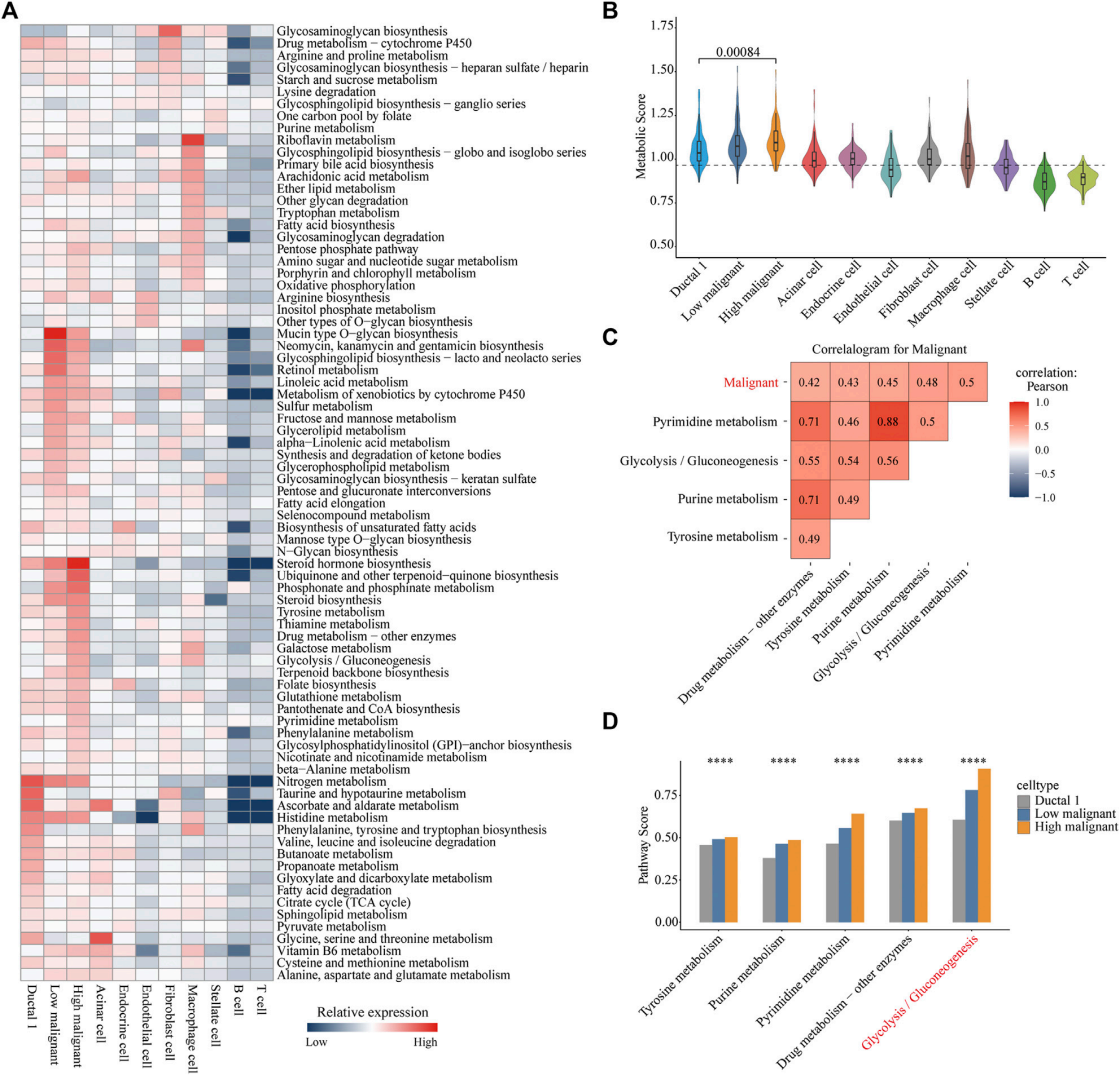
Metabolic reprogramming in highly malignant cell subpopulations. **(A)** Heatmap of expression demonstrating the abundance of Kyoto Encyclopedia of Genes and Genomes (KEGG) metabolic pathways in different cell types in pancreatic ductal adenocarcinoma. **(B)** Violin plots for metabolic score of each cell type. Wilcoxon rank test, *p* = 0.00084. **(C)** Correlation heat map (Pearson correlation) of the metabolic pathways (top five) with the highest correlation to malignant scores in all carcinoma cells. Red, positive correlation; blue, negative correlation; white, no correlation. **(D)** Bar chart is shown with the pathway scores of five metabolic pathways in three cell types, and *p*-values were calculated using ANOVA analysis. Ductal 1 cell, gray bar chart; lowly malignant, blue bar chart; highly malignant, orange bar chart. ****p < 0.0001.

We referenced a public method to quantify the metabolic pathways of different cell types. This allowed further investigation of the variation and overall features of metabolic pathways among different cell types, especially between the highly and lowly malignant cell subpopulations. Distinct cell types exhibited distinguished metabolic profiles. Among immune cells, macrophages demonstrated the highest metabolic activity. Riboflavin metabolism is significantly upregulated in macrophages. In fibroblasts, glycosaminoglycan biosynthesis was significantly upregulated. Strikingly, the subpopulation of highly malignant cells illustrated the highest metabolic activity among all cell types. Moreover, lowly malignant cells had a more active metabolic pathway than that of ductal one cells. This suggested that the metabolic activity was correlated with the malignant progression of tumor cells (Figures 2A, B). Of all 79 metabolic pathways, 44 were highly expressed in highly malignant cell subpopulations. The categories of overexpressed metabolic pathways were mainly concentrated in carbohydrate metabolism, metabolism of cofactors and vitamins, glycan biosynthesis, and metabolism as well as the lipid metabolism (Supplementary Figure S2B).

To further reveal the significant metabolic pathways of highly malignant cell subpopulations, we performed a Pearson correlation analysis of all metabolic pathway scores with cellular malignant scores. Pyrimidine metabolism, glycolysis/gluconeogenesis, purine metabolism, tyrosine metabolism, and drug metabolism-other enzymes demonstrated a significant positive correlation with the malignancy of tumor cells (Figure 2C and Supplementary Figure S2C), indicating that these five metabolic pathways may be associated with the malignant progression of PDAC. Subsequently, we compared the expression of these five metabolic pathways in ductal 1 cells and lowly and highly malignant cells. Glycolysis was found to be most significantly elevated in highly malignant tumor cells (Figure 2D). These analyses suggested that enhanced glycolysis is the main metabolic feature in highly malignant cell subpopulations.

### Glycolytic Marker Genes of Highly Malignant Cell Subpopulations

**FIGURE 3:**
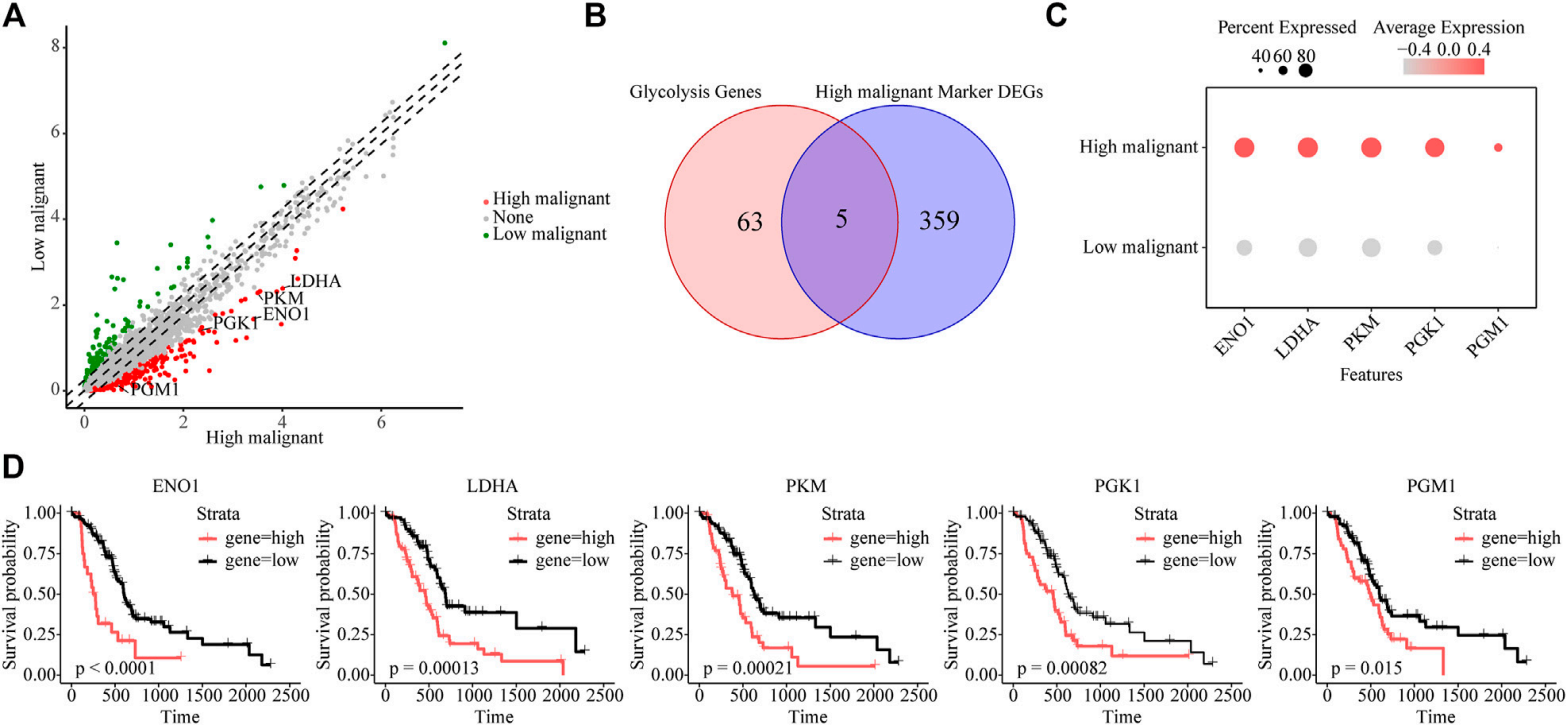
Identifying glycolytic marker genes in highly malignant cell subpopulations. **(A)** Scatter plot of differentially expressed genes. Green, lowly malignant marker gene; red, highly malignant marker gene. **(B)** Venn diagram showing five marker glycolytic genes among the highly malignant cell marker genes. **(C)** Dot plot to show the expression differences of five glycolytic marker genes ranked from highest to lowest. **(D)** The association of five marker glycolytic genes with survival based on TCGA data analysis.

To further reveal key genes of glycolysis during malignant progression of PDAC tumor cells, we compared highly and lowly malignant cells by using |logfc| > 1 and *p*-value <0.01 and found a total of 364 genes to be significantly upregulated in highly malignant cells (Figure 3A and Supplementary Table S3). Among these 364 genes, glycolytic genes were identified, including *EN O 1*, *LDHA*, *PKM*, *PGK1*, and *PGM1* (Figures 3B, C). In addition, 16 of these 364 genes belong to the five malignant-related metabolic pathways identified previously. The Protein–Protein Interaction (PPI) network confirmed the centrality of glycolytic marker genes among malignancy-associated metabolic genes (Supplementary Figure S4A). Furthermore, we attempted to analyze the association of five glycolytic genes with patient prognosis. High expressions of *EN O 1* (*p* < 0.0001), *LDHA* (*p* = 0.00013), *PKM* (*p* = 0.00021), *PGK1* (*p* = 0.00082), and *PGM1* (*p* = 0.015) were related to significantly poorer survival in patients with PDAC (Figure 3D), indicating the five genes can be used as glycolytic marker for highly malignant cell subpopulations.

### mRNA and Protein Expression Profiles of Five Glycolytic Marker Genes in PDAC

**FIGURE 4:**
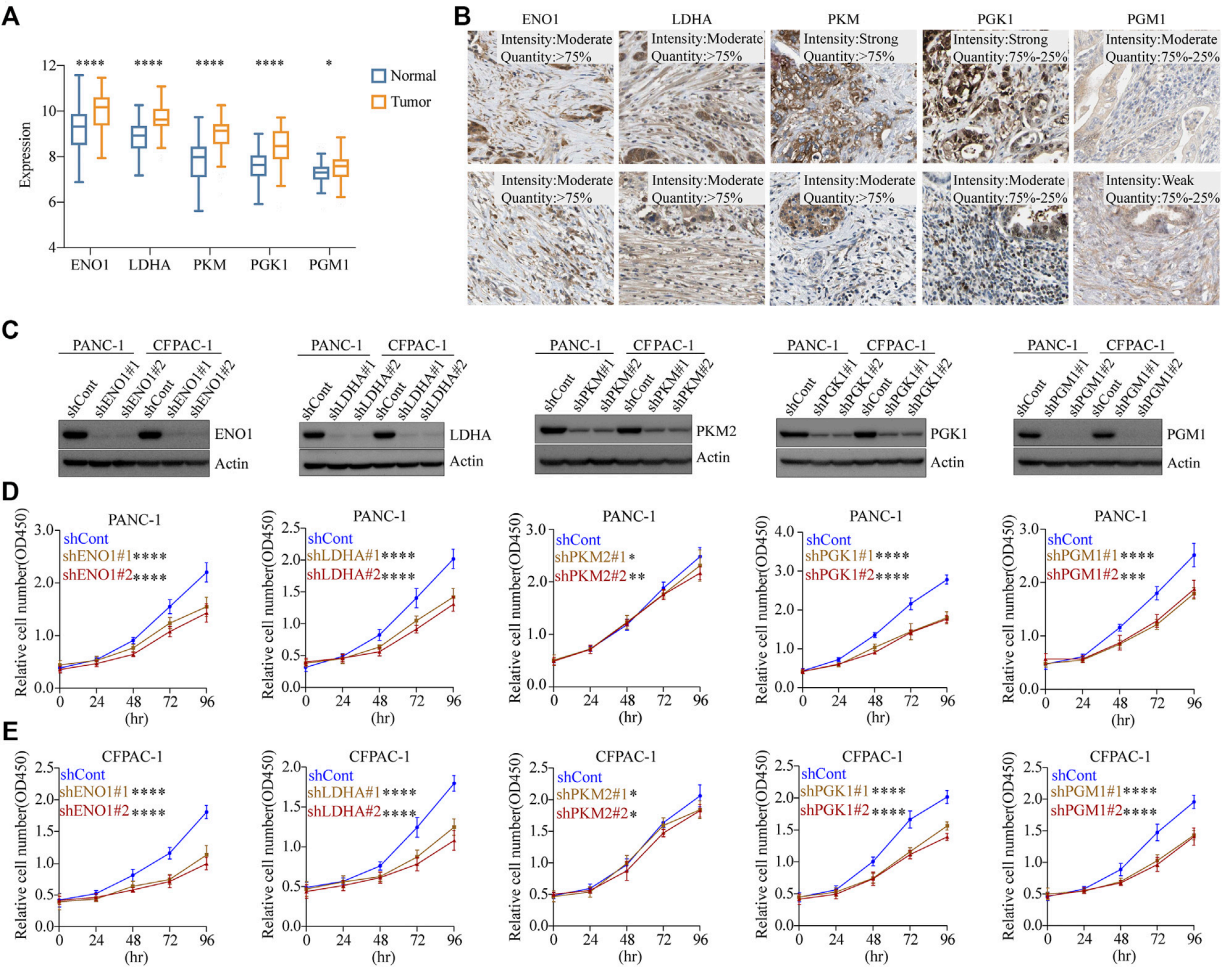
Glycolytic marker genes involved in the malignant progression of PDAC. **(A)** Expression levels of five glycolytic marker genes between tumor and paired normal tissues in the GSE62452 cohort. Blue, normal; orange, tumor. **(B)** Expression levels of *EN O 1*, *LDHA*, *PKM*, *PGK1*, and *PGM1* protein in 10 patients with PDAC from the HPA database. Scale bar: 20 µm. **(C)** PANC-1 cells and CFPAC-1 cells were transfected with sh-control, sh-ENO1, sh-LDHA, sh-PKM2, sh-PGK1, and sh-PGM1. Validation of knockdown efficiency using Western blot. **(D)** PANC-1 cells viability was measured by CCK8 assay after knockdown of five glycolytic marker genes, respectively. **(E)** CFPAC-1 cells viability was measured by CCK8 assay after knockdown of five glycolytic marker genes, respectively. *p < 0.05; **p < 0.01; ***p < 0.001; ****p < 0.0001.

To investigate the mRNA expression levels of the five glycolytic marker genes in PDAC, datasets from the Gene Expression Omnibus (GEO) database were used for further analysis. The GSE62452 dataset containing a total of 130 samples was used, including both tumor and paired normal biopsy samples. After normalizing the expression profile, mRNA expression levels of the five glycolytic marker genes in PDAC were significantly elevated compared with paired normal biopsy tissues (Figure 4A). Furthermore, the mRNA expression of these five genes was similarly studied in the TCGA cohort. Similarly, elevated mRNA levels of the five glycolytic marker genes were observed in tumor tissues. This finding was observed in the analysis that was performed using the TCGA program and the GTEx program (Supplementary Figure S4B). The subsequent step was to examine the positive expression of the five glycolytic marker genes in PDAC using immunohistochemical (IHC) analysis using the HPA. The mRNA and protein expression of the five genes were all overexpressed in PDAC (Figure 4B). These results are consistent with those of previous analysis of scRNA-seq data.

### Glycolytic Marker Genes Involved in the Malignant Progression of PDAC Cells

To investigate the effect of glycolytic marker genes in PDAC cells, we silenced the five glycolytic marker genes separately in PANC-1 and CFPAC-1 cell lines. Two unique silencing sites were used for each gene. Western blot indicated a significant decrease in the expression of *EN O 1*, *LDHA*, *PKM2*, *PGK1*, and *PGM1* compared with the control cells (Figure 4C). The absorbance of each group at different time points was measured using the CCK8 proliferation assay. As demonstrated, in both PANC-1 and CFPAC-1 cell lines, silencing of the glycolytic marker genes decreased the proliferative capacity compared with the shCont group (Figures 4D, E). In normal pancreatic ductal cell line HPDE, the knockdown of these five glycolytic genes did not have as marked an effect on proliferation rates as in tumor cells (Supplementary Figure S3A, B). Collectively, these data confirmed the contribution of the five glycolytic marker genes in the malignant progression of PDAC.

### Clinical Features of Highly Malignant Cell Subpopulations

**FIGURE 5:**
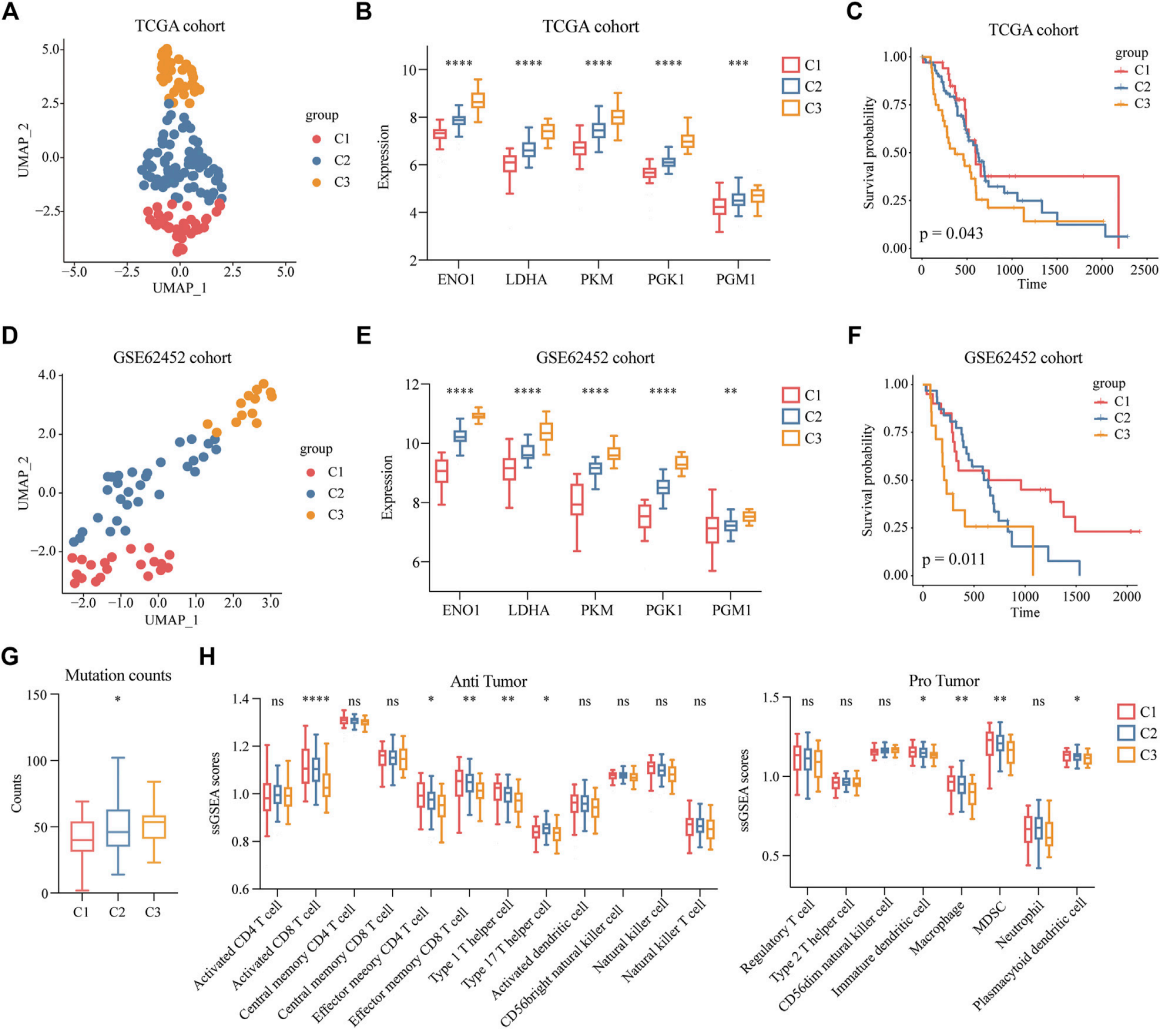
Clinical features of patients with highly malignant cell subpopulation. **(A)** UMAP plot of five glycolytic marker genes expression values based on the TCGA cohort and divided into clusters C1, C2, and C3. **(B)** Expression levels of five glycolytic marker genes in groups C1 (low expression), C2 (median expression), and C3 (high expression) among TCGA cohort. **(C)** Overall survival of C1, C2, and C3 groups in TCGA cohort. **(D)** UMAP plot of five glycolytic marker genes expression values based on the GSE62452 cohort and divided into clusters C1, C2, and C3. **(E)** Expression levels of five glycolytic marker genes in groups C1 (low expression), C2 (median expression), and C3 (high expression) among the GSE62452 cohort. **(F)** Overall survival of C1, C2, and C3 groups in GSE62452 cohort. **(G)** Comparisons of the counts of mutations in groups C1, C2, and C3. **(H)** Infiltration scores of anti-tumor and pro-tumor immune cells in C1, C2, and C3 groups. *p < 0.05; **p < 0.01; ***p < 0.001; ****p < 0.0001.

To determine the clinical features of the highly malignant cell subpopulation, we obtained the expression profiles of the five marker genes from TCGA (n = 146) and used UMAP to reduce the dimensionality (Figure 5A). A linear ascending characteristic was demonstrated for the five marker genes, and the patients were divided into C1, C2, and C3 groups according to the interquartile cut-off. (Figure 5B). The five glycolytic marker genes had the highest expression in the C3 subtype and revealed significant prognostic differences, with the C3 subtype having the lowest survival rate (*p* = 0.043) (Figure 5C). A similar increasing trend and survival difference were surprisingly maintained in the GSE62452 (n = 65) cohort (*p* = 0.011) (Figures 5D–F). As illustrated in Table 1, the expression of glycolytic marker genes was correlated with pathological grade only in patients with PDAC. Similar results were observed in the GSE62452 cohort (Table 2).

**TABLE 1:** Correlation of C1, C2, and C3 subtypes of clinicopathological features in patients with PDAC from TCGA cohort.

Parameter	—	TCGA cohort			*p*-value
—	—	C1(*N* = 37)	C2(*N* = 72)	C3(*N* = 37)	—
Age	<65 years	14 (9.59%)	30 (20.55%)	21 (14.38%)	0.21
—	≥65 years	23 (15.75%)	42 (28.77%)	16 (10.96%)	—
Gender	Male	22 (15.07%)	38 (26.03%)	18 (12.33%)	0.64
—	Female	15 (10.27%)	34 (23.29%)	19 (13.01%)	—
Stage	Stage I	2 (1.37%)	8 (5.48%)	2 (1.37%)	0.66
—	Stage II	32 (21.92%)	61 (41.78%)	34 (23.29%)	—
—	Stage III	1 (0.68%)	2 (1.37%)	0 (0.0e+0%)	—
—	Stage IV	1 (0.68%)	1 (0.68%)	1 (0.68%)	—
Grade	G1	9 (6.16%)	11 (7.53%)	1 (0.68%)	**0.04**
—	G2	17 (11.64%)	45 (30.82%)	21 (14.38%)	—
—	G3	10 (6.85%)	16 (10.96%)	15 (10.27%)	—
—	G4	1 (0.68%)	0 (0.0e+0%)	0 (0.0e+0%)	—
T	T1	1 (0.68%)	2 (1.37%)	1 (0.68%)	0.65
—	T2	4 (2.74%)	10 (6.85%)	2 (1.37%)	—
—	T3	30 (20.55%)	58 (39.73%)	34 (23.29%)	—
—	T4	1 (0.68%)	2 (1.37%)	0 (0.0e+0%)	—
—	NA	1 (0.68%)	0 (0.0e+0%)	0 (0.0e+0%)	—
N	N0	9 (6.16%)	21 (14.38%)	7 (4.79%)	0.38
—	N1	28 (19.18%)	51 (34.93%)	29 (19.86%)	—
—	NX	0 (0.0e+0%)	0 (0.0e+0%)	1 (0.68%)	—
M	M0	17 (11.64%)	33 (22.60%)	19 (13.01%)	0.95
—	M1	1 (0.68%)	1 (0.68%)	1 (0.68%)	—
—	MX	19 (13.01%)	38 (26.03%)	17 (11.64%)	—

**TABLE 2:** Correlation of C1, C2, and C3 subtypes of clinicopathological features in patients with PDAC from GSE62452 cohort.

Parameter	—	GSE62452 cohort			*p*-value
—	—	C1(*N* = 20)	C2(*N* = 31)	C3(*N* = 14)	—
Stage	Stage I	1 (1.54%)	1 (1.54%)	2 (3.08%)	0.47
—	Stage II	23 (35.38%)	7 (10.77%)	14 (21.54%)	—
—	Stage III	5 (7.69%)	3 (4.62%)	2 (3.08%)	—
—	Stage IV	2 (3.08%)	3 (4.62%)	1 (1.54%)	—
—	Stage >II	0 (0.0e + 0%)	0 (0.0e + 0%)	1 (1.54%)	—
Grade	G1	0 (0.0e + 0%)	0 (0.0e + 0%)	2 (3.08%)	**0.02**
—	G2	16 (24.62%)	3 (4.62%)	13 (20.00%)	—
—	G3	15 (23.08%)	10 (15.38%)	4 (6.15%)	—
—	G4	0 (0.0e + 0%)	1 (1.54%)	0 (0.0e + 0%)	—
—	Gx	0 (0.0e + 0%)	0 (0.0e+0%)	1 (1.54%)	—

Furthermore, to investigate the mutation and immune features of the C3 subtype, mutation data of 146 patients from the TCGA database were analyzed. In terms of the count of mutations, the C3 subtype exhibited the highest number of mutations (Figure 5G). Specifically, the top 10 genes with the highest mutation rate in the C3 subtype were *KRAS*, *TP53*, *CKDN2A*, *CKDN2A-DT*, *CKDN2B*, *SMAD4*, *MTAP*, *RN7SL151P*, *KLHL9*, and *IFNE*, which reflected the excessive mutational burden of the C3 subtype (Supplementary Figure S4C and Supplementary Table S4). Furthermore, we classified immune cells as anti-tumor and pro-tumor based on the known functions of the cells. The immune infiltration scores were calculated using the gene signatures of immune cells. We observed a significant decrease in the C3 subtypes of multiple anti-tumor immune cells, including activated CD8 T cells, effector memory CD4 T cells, effector memory CD8 T cells, type 1 T helper cells, and type 17 T helper cells. Remarkably, we observed a low level of infiltration of pro-tumor immune cells in the C3 subtype, including immature dendritic cells, macrophages, myeloid-derived suppressor cells (MDSCs), and plasmacytoid dendritic cells (Figure 5H). This finding indicated that nearly all immune cells were suppressed in the C3 subtype.

### GHS Scores Constructed by Glycolysis Marker Genes Is an Independent Prognostic Factor

**FIGURE 6:**
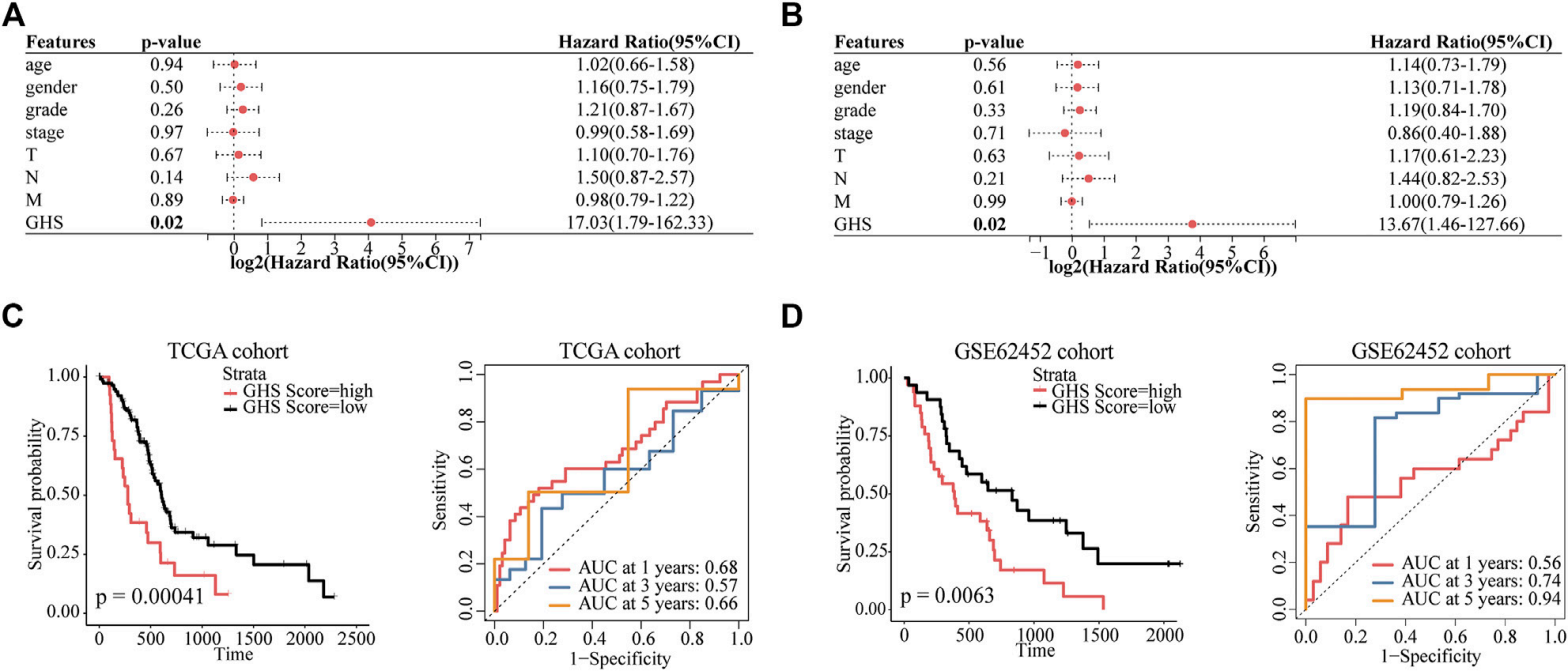
GHS score is an independent prognostic factor for PDAC patients. **(A)** Forest plot of GHS related to overall survival in univariate Cox regression analysis. **(B)** Forest plot of GHS related to overall survival in multivariate Cox regression analysis. **(C)** TCGA cohort overall survival (OS) curves for high and low GHS score groups (left) (time-dependent ROC curves for predicted 1-/3-/5-year overall survival by GHS score (right). **(D)** GSE62452 cohort overall survival (OS) curves for high and low GHS score groups (left) (time-dependent ROC curves for predicted 1-/3-/5-year overall survival by GHS score (right).

To evaluate the value of five glycolytic marker genes as prognostic markers in patients with PDAC, by using the above formula, five glycolytic marker genes were integrated to establish the GHS score (Supplementary Figure S4D). Univariate and multivariate Cox regression analyses were performed to further test the independent role of GHS score on other clinical-pathological variables. Our results revealed that the GHS score was an independent prognostic factor for patients with PDAC (HR = 13.67; 95% confidence interval (CI) 1.46–127.66; *p* = 0.02; Figures 6A, B).

Furthermore, we tested the reliability of GHS via survival analysis and ROC analyses. In the TCGA cohort, Kaplan–Meier survival analysis revealed a significantly worse prognosis in the higher GHS scores group (*p* = 0.00041). GHS scores had significant prognostic value after 1 (area under the curve [AUC] = 0.68), 3 (AUC = 0.57), and 5 years (AUC = 0.66; Figure 6C). Likewise, the same reliable prognostic value was obtained in the GSE62452 cohort. The higher GHS scores group exhibited a lower survival rate (*p* = 0.0063). The GHS scores of time-dependent ROC analysis at 1, 3, and 5 years were 0.56, 0.74, and 0.94, respectively (Figure 6D), indicating that the GHS score could be used to predict the prognosis of patients with PDAC.

## Discussion

In this study, we combined scRNA-seq, bulk RNA-seq, and clinical data for bioinformatics analysis. A subpopulation of highly malignant cells with a high degree of glycolysis as the main feature was identified in patients with PDAC. Five glycolytic marker genes including *EN O 1*, *LDHA*, *PKM*, *PGK1*, and *PGM1* were associated with the malignant progression of PDAC, and a GHS score is proposed, which could also be used as biomarkers to determine the metabolic subtype and prognosis of patients.

PDAC cells adopt different metabolic pathways to meet their growth requirements. Targeting the signature metabolic pathway of the tumor is expected to be a new anti-cancer strategy (Martinez-Outschoorn et al., 2017); however, the poor prognosis of patients with pancreatic cancer is not greatly improved after the use of metabolic therapy (Baron et al., 2021). This may be attributed to the differences in metabolic subtypes.

It was reported that an extensive metabolite analysis of several PDAC cell lines identified three metabolic subtypes exhibiting different metabolite profiles related to glycolysis, lipogenesis, and oxidation–reduction pathways (Daemen et al., 2015). In addition, recent studies have established four metabolic subgroups, namely, quiescent, glycolytic, cholesterogenic, and mixed, using genomic and transcriptomic data from 325 patients with PDAC (Karasinska et al., 2020). The glycolytic subtype had the worst survival outcome, while cholesterogenic subtype had the longest survival time. However, no suitable markers are available to identify the metabolic subtypes of patients.

Previous studies mostly performed comprehensive genomic analyses by using bulk RNA-seq (Espiau-Romera et al., 2020). In contrast to classical bulk RNA-seq, scRNA-seq allows the accurate discrimination between different cell types in bulk tissue, broadening the understanding of tumor biology (Stuart and Satija, 2019). However, scRNA-seq data are often extremely noisy due to the low numbers of mRNA detectable in individual cells and the large intercellular differences (Kim et al., 2020). These limitations make it difficult to distinguish some lowly expressed and non-expressed genes of interest. Notably, our metabolic pathway analysis based on the single-cell resolution has very similar results to that based on pseudo-bulk data (Supplementary Figure S5A). The analysis based on pseudo-bulk data demonstrates some differences in low expression metabolic pathways.

Although scRNA-seq has advanced our understanding of tumor heterogeneity significantly, direct observation of cellular metabolism at the single-cell level is challenging. Metabolic gene expression levels are not equivalent to metabolic fluxes, but they can predict metabolic fluxes to some extent (Bidkhori et al., 2018). Sophisticated bioinformatics analysis methods are expected to make up for these limitations. Damiani et al. developed a computational framework for transforming single-cell transcriptomes into single-cell fluxomic (Damiani et al., 2019). However, the accuracy of this calculation framework needs to be further explored.

All five glycolytic marker genes expressed in pancreatic cancer were associated with the poor progression of PDAC. Alpha-enolase (*EN O 1*), one of the three enolase isozymes, participates in the adhesion, invasion, and metastasis of PDAC by controlling integrin expression (Principe et al., 2017). Lactate dehydrogenase A (*LDHA*) is a cytoplasmic enzyme, and overexpression of *LDHA* promotes PDAC proliferation and invasion *in vitro* by regulating phosphorylation of AMPK and mTOR (Cheng et al., 2021). Inhibition of *LDHA* has no significant toxic effects on normal tissues; therefore, *LDHA* may serve as a promising target for tumor therapy. Pyruvate kinase (*PKM*) has two isomers, namely, *PKM1* and *PKM2*, and exists as *PKM2* in tumor tissues. When *PKM2* is knocked down, PDAC cells can temporarily provide pyruvate via cysteine catabolism to meet cell growth requirements (Yu et al., 2019). In addition, *PKM*2 has been reported to be involved in PDAC invasion and metastasis through phosphorylation of PAK2 (Cheng et al., 2018). Phosphoglycerate kinase 1 (*PGK1*) is secreted by tumor cells and plays a role in coordinating glycolysis and the TCA cycle during tumorigenesis (Li et al., 2016). Phosphoglucomutase 1 (*PGM1*) is mainly involved in glucose catabolism and synthesis and enhances the proliferation and metastasis of gastric cancer cells (Cao et al., 2021). However, *PGM1* is involved in tumor suppression in liver cancer (Jin et al., 2018). Therefore, *PGM1* may play distinct roles in different cancers. To the best of our knowledge, the study of *PGM1* in PDAC has not yet been reported, and our study revealed that *PGM1* is a marker of malignancy in PDAC and plays an important role in PDAC glycolysis. Glycolysis is a vital component of cellular metabolism, and it plays an important role in maintaining cell growth, even in normal cells (Lunt and Vander Heiden, 2011). Here, we found that the effect of the five glycolytic genes on the proliferation rate of pancreatic normal ductal cells was not as significant as in tumor cells.

In this study, the GHS score was proposed from five glycolytic highly malignant-related genes. The effectiveness of the GHS score was validated in the TCGA and GSE62452 cohorts, and the prognostic value of the GHS score for PDAC patients should further be verified in prospective clinical studies. The biological mechanisms of the five genes, which contribute to the progression of PDAC, require further investigations.

Moreover, metabolic reprogramming during the development of malignant tumors causes alterations in the tumor microenvironment. Recent studies have suggested three major immune subtypes of tumors as follows: immune infiltrative, immune exclusive, and immune desert (Desbois et al., 2020). It is believed that excessive glycolysis in tumors suppresses anti-tumor immunity (Cascone et al., 2018), while our study demonstrated that the high glycolytic subtype in PDAC exhibited a more immune desert profile. Tumor cells affect the nutrition of almost all immune cells, and further investigation is required for confirmation.

## Conclusion

We have revealed a highly malignant cell subpopulation in PDAC that exhibited a high glycolysis rate as the main feature and obtained five glycolytic marker genes with their biological functions that are closely related to the PDAC development. These genes can be used as markers to determine the metabolic subtype and prognosis of patients with PDAC.

## Data Availability

The original contributions presented in the study are included in the article/Supplementary Material, further inquiries can be directed to the corresponding authors.
