# Supplementary material for: Single-Cell Transcriptome Reveals the Metabolic and Clinical Features of a Highly Malignant Cell Subpopulation in Pancreatic Ductal Adenocarcinoma
Source: Front Cell Dev Biol. 2022 Feb 18;10:798165. doi: 10.3389/fcell.2022.798165 (PMC8894596; doi:10.3389/fcell.2022.798165)
Supplement: Supplementary file 5 [file DataSheet1.docx]

Supplementary Material

## Supplementary Figures


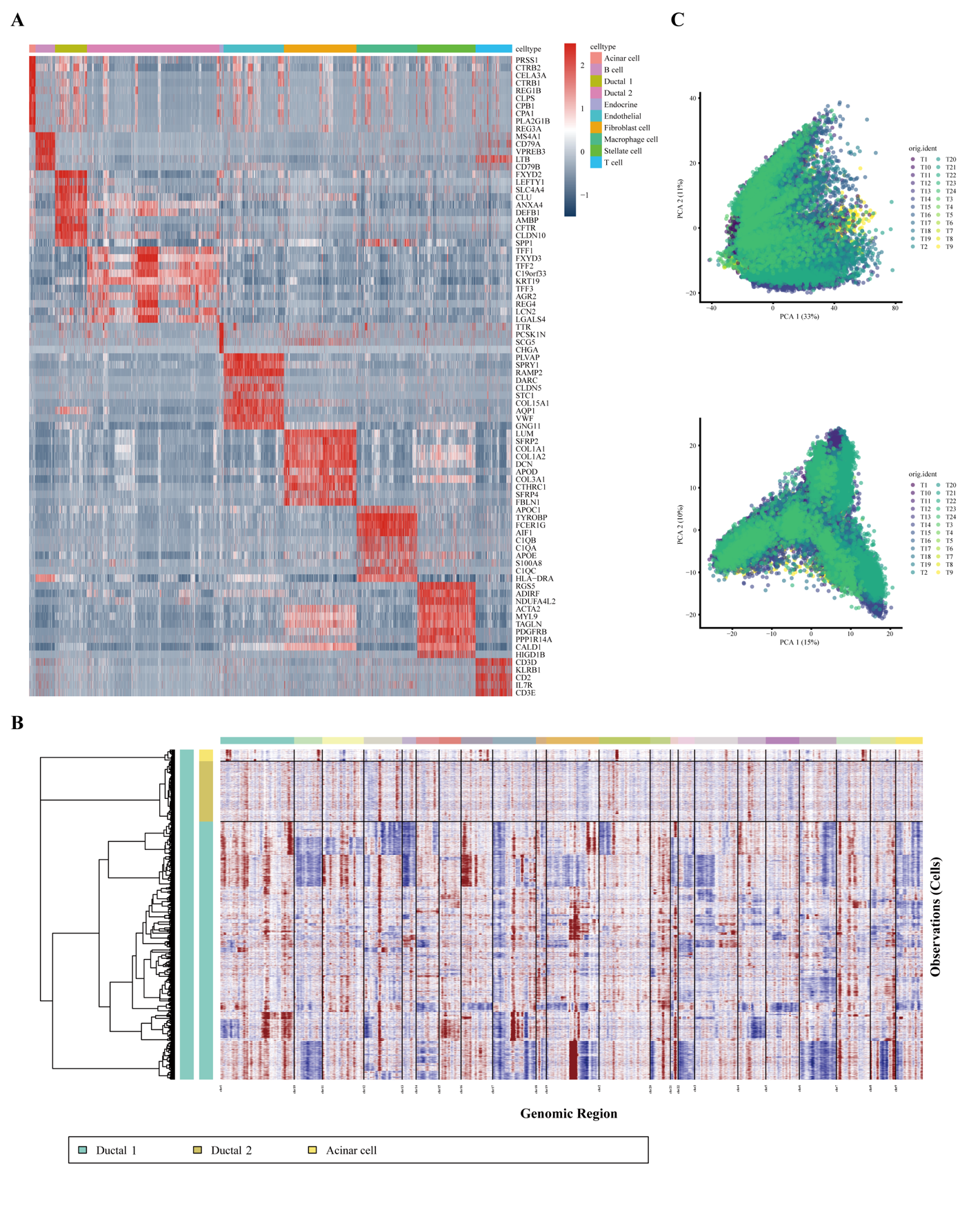


**Supplementary Figure 1.** **(A)** Heatmap showing the expression pattern of marker genes for each cell type with selected markers (red = high expression; blue = low expression). **(B)** CNVs of Ductal 2, Ductal 1 and Acinar were annotated by chromosome arms, where CNV status were inferred by inferCNV. **(C)** Original data PCA and standardized data PCA comparison (Above=raw data, below=normalized data).

**
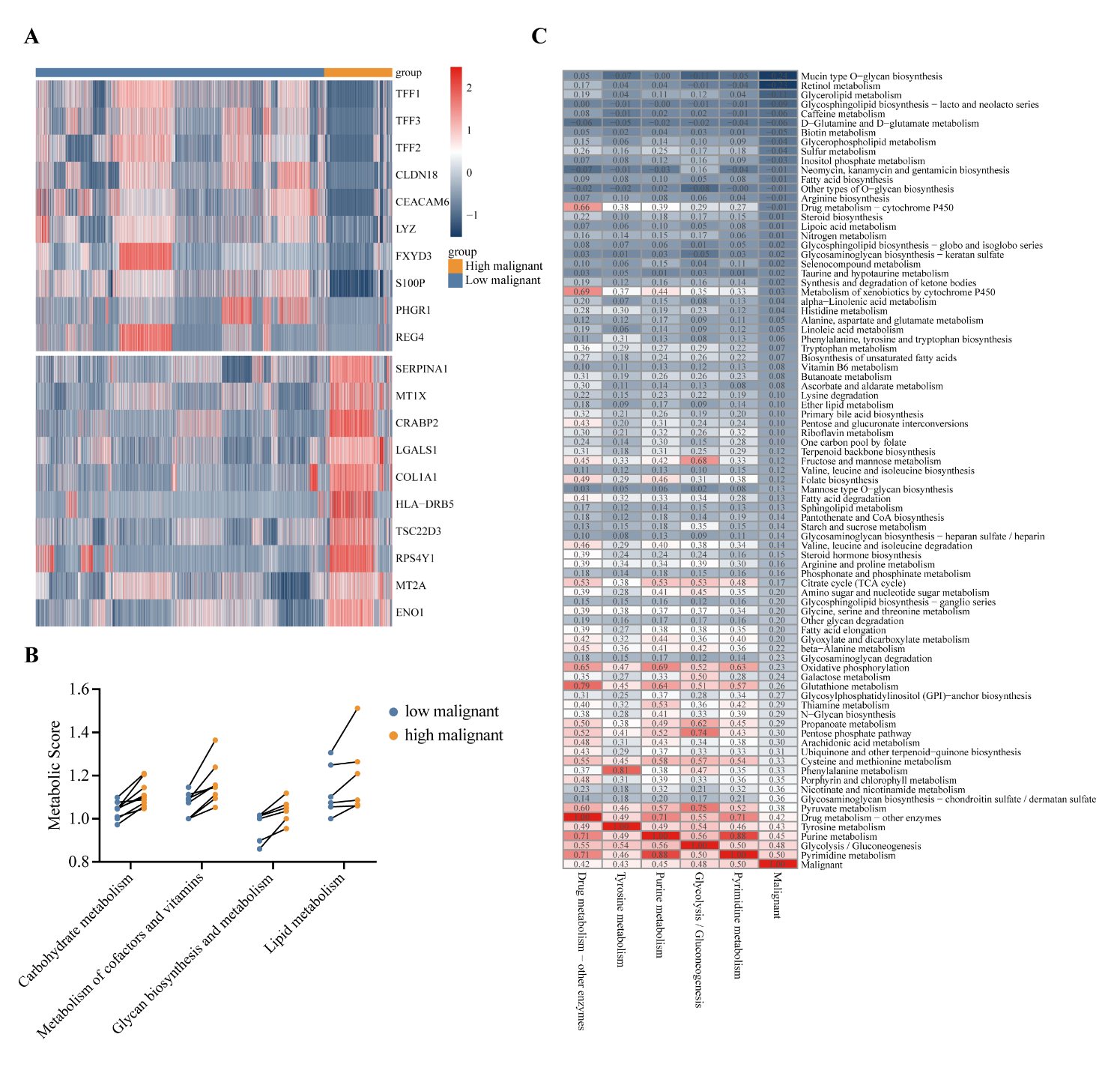
**

**Supplementary Figure 2. (A)** A heatmap of marker genes for High malignant and low malignant cells (top10). **(B)** Scatter plot illustrating the main types of metabolic pathways upregulated in a subpopulation of highly malignant cells. **(C)** The Correlation heatmap of all Metabolic Pathways and Malignancy in PDAC Cancer Cells.

**
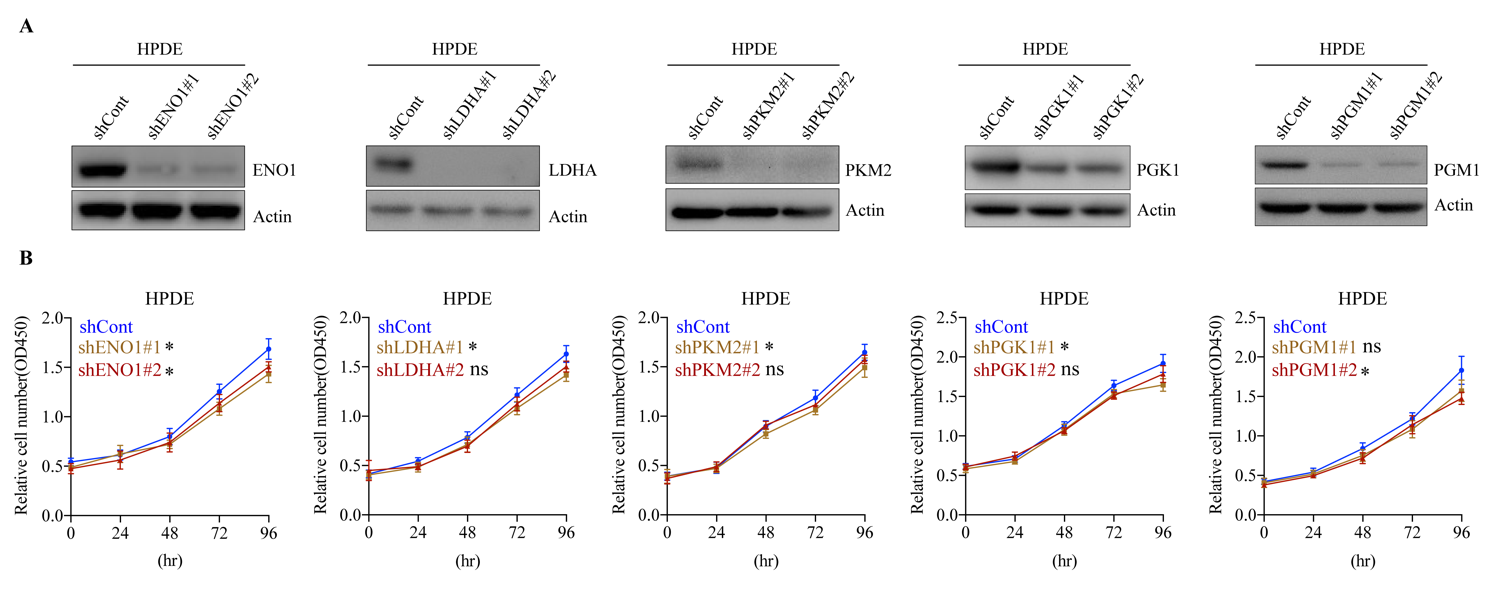
**

**Supplementary Figure 3. (A)** HPDE cells were transfected with sh-control, sh-ENO1, sh-LDHA, sh-PKM2, sh-PGK1 and sh-PGM1. Validation of knockdown efficiency using Western blot. **(B)** HPDE cells viability was measured by CCK8 assay after knockdown of five glycolytic marker genes, respectively. **p* < 0.05; ***p* < 0.01; ****p* < 0.001; *****p* < 0.0001.

**
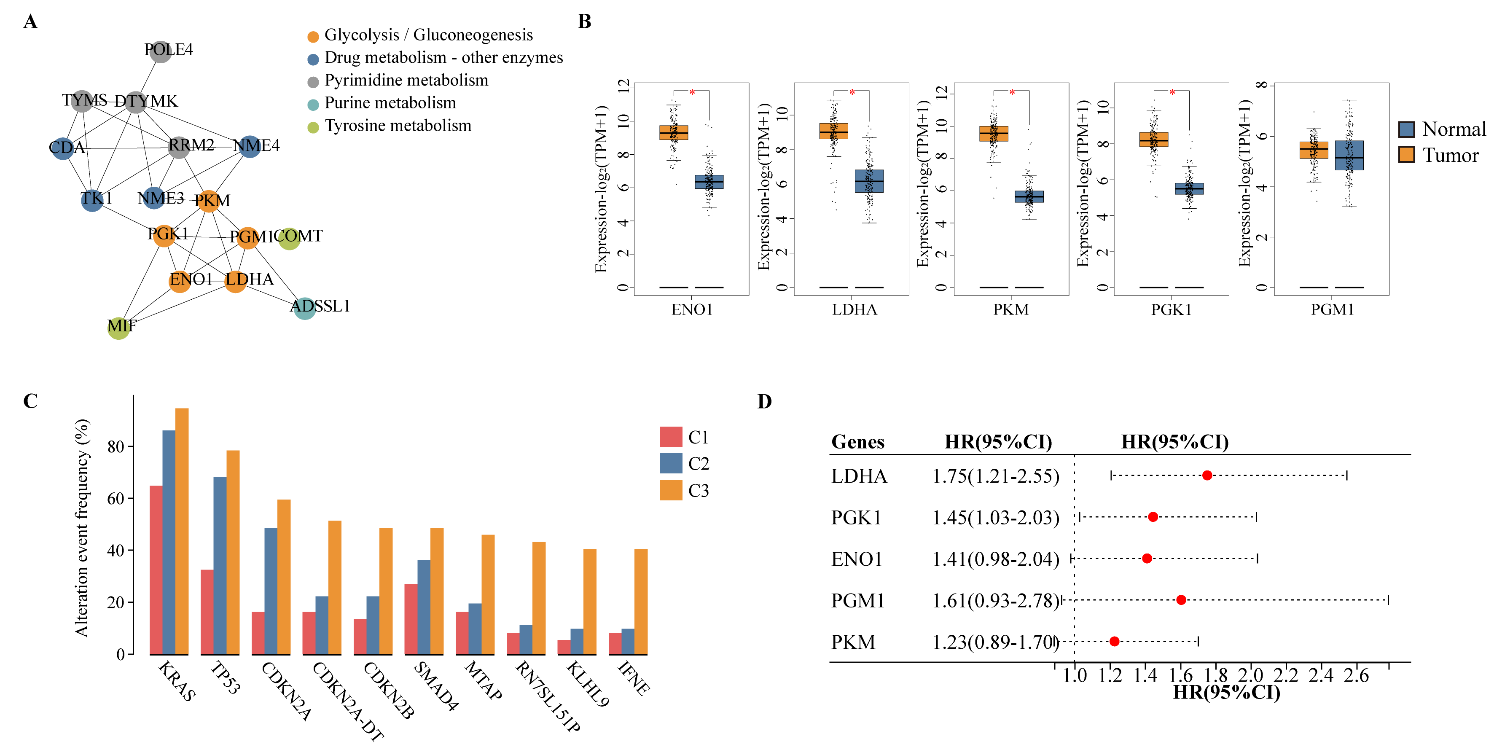
**

**Supplementary Figure 4. (A)** PPI network graph of 16 malignancy-associated metabolic genes. **(B)** The mRNA expression of *ENO1*, *LDHA*, *PKM*, *PGK1* and *PGM1* in pancreatic cancer and normal tissues as measured in the TCGA program and GTEx program. **(C)** Top 10 genes with high mutation rates in C3 subtypes. **(D)** Hazard factors for *ENO1*, *LDHA*, *PKM*, *PGK1* and *PGM1*.


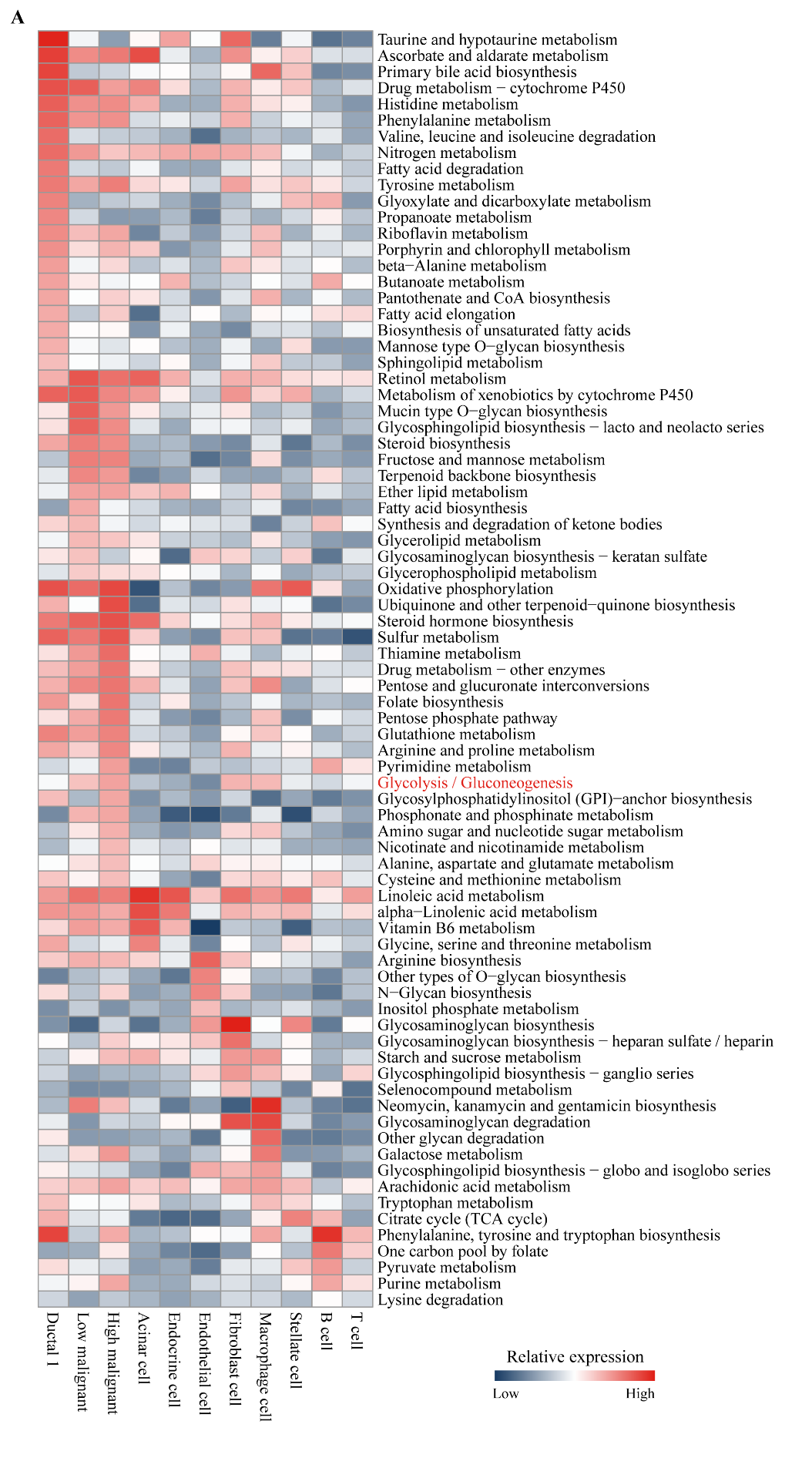


**Supplementary Figure 5. (A)** Heatmap of expression demonstrating the abundance of KEGG metabolic pathways in different cell types based on the pseudo-bulk dataset.
